# A case report on the clinical management strategy for renal arteriovenous fistula and severe abdominal hemorrhage complicating ultrasound-guided renal puncture biopsy

**DOI:** 10.3389/fphys.2026.1753385

**Published:** 2026-04-29

**Authors:** Wang Xiangdong

**Affiliations:** Department of Ultrasound Medicine, Shenzhen Yantian District People’s Hospital, Shenzhen, China

**Keywords:** arteriovenous fistula, color Doppler ultrasound, complications, postoperative bleeding, renal biopsy, ultrasound-guided intervention

## Abstract

Renal puncture biopsy is widely used in the pathological diagnosis of renal diseases, but it is accompanied by the risk of bleeding complications. Severe bleeding can lead to hemorrhagic shock, arteriovenous fistula, and even be life threatening. This paper reports a 58 year old male patient who developed severe perirenal hematoma and renal arteriovenous fistula after ultrasound guided renal puncture biopsy, resulting in hemorrhagic shock. After emergency interventional embolization, the fistula was successfully closed, and the patient recovered well. This case suggests that clinicians should strengthen perioperative evaluation and monitoring, identify complications early, and adopt timely and effective intervention measures to improve the safety of patients.

## Brief medical history

A 58-year-old male patient was admitted for “foamy urine for over 3 months and elevated creatinine for 3 days.” On admission, physical examination revealed: temperature, 36.7°C; pulse, 80 bpm; resting heart rate, 20 bpm; and blood pressure (BP), 155/86 mmHg. Urinalysis revealed proteinuria (3+), negative occult blood, negative red blood cells, serum creatinine of 198 μmol/L, and uric acid of 660 μmol/L. After 15 days of hospitalization, the creatinine levels progressively increased. The patient consented to a renal biopsy. An ultrasound-guided renal biopsy was performed in the Ultrasound Imaging Department. The patient was positioned prone with the abdominal lower costal margin elevated 5–10 cm, both upper limbs placed at the sides, and the head turned to one side. The patient was instructed to breathe calmly. The puncture site was localized using color Doppler ultrasound. A 16-G tissue biopsy gun was advanced through the guidewire, extracting a renal tissue sample approximately 1.2 cm in length. Two specimens were obtained, with satisfactory tissue acquisition.

At 1 h after the puncture, the patient reported excruciating pain in the right abdomen. Physical examination revealed: alert and oriented, expression of distress, right abdominal distension, and marked tenderness. Electronic BP was 130/81 mmHg. Urinary system ultrasound revealed right renal lower pole hematoma and right abdominal hematoma formation. Urgent blood tests were ordered, blood transfusion was prepared, and a critical condition was declared. After 2 h, the patient’s BP dropped to 79/50 mmHg, and his heart rate was 75 bpm. Hemorrhagic shock was suspected. Emergency fluid resuscitation stabilized the BP above 100/60 mmHg. Immediate hospital-wide consultation was requested involving Cardiology, Thoracic Surgery, Emergency Medicine, and Urology. Under local anesthesia in the interventional catheterization laboratory, a 6-F vascular sheath was placed via the Seldinger technique through a right radial artery puncture. A contrast catheter was advanced for right renal artery angiography, which revealed an arteriovenous fistula in the posterior branch of the middle segment of the right renal artery. Guided by the angiographic catheter and supported by a Rengade microcatheter, a FATHOM-16 steerable guidewire was advanced to the posterior branch of the middle segment of the right renal artery. Contrast injection further confirmed the lesion location. A 4-mm × 8-cm releasable caged coil was advanced through the microcatheter and deployed in the middle posterior branch of the right renal artery. Follow-up angiography showed no contrast leakage. The procedure concluded with stable vital signs. Discharge at diagnosis was as follows: acute renal failure and post-procedural renal hemorrhage: post-renal puncture hemorrhage, post-renal puncture hematoma, renal arteriovenous (AV) fistula, and amyloid light-chain (AL)-type renal amyloidosis. Follow-up through August 2023 showed the patient’s recovery progressing well.

**Figure 1:**
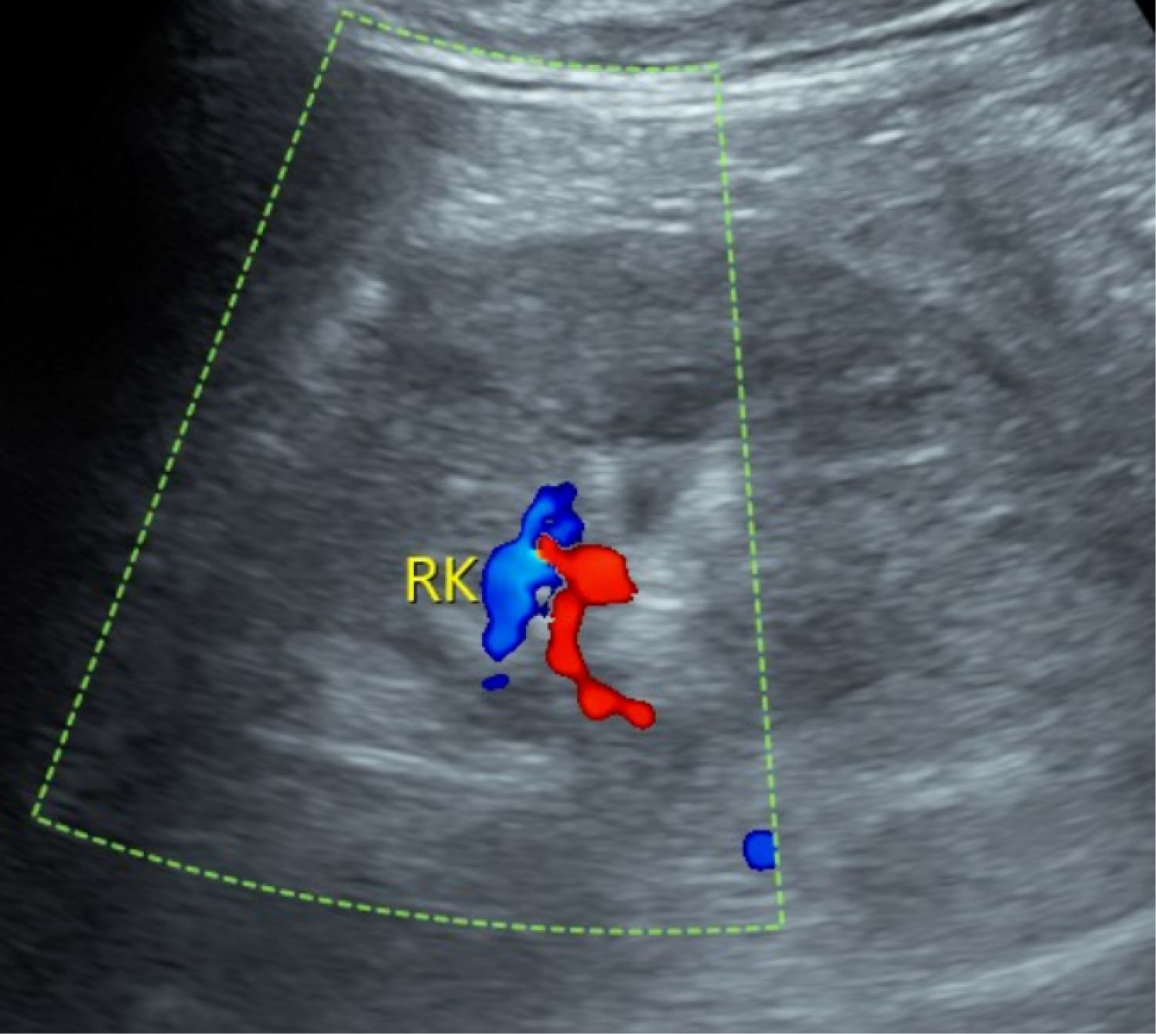
Color Doppler ultrasound image of the right kidney prior to puncture.

**Figure 2:**
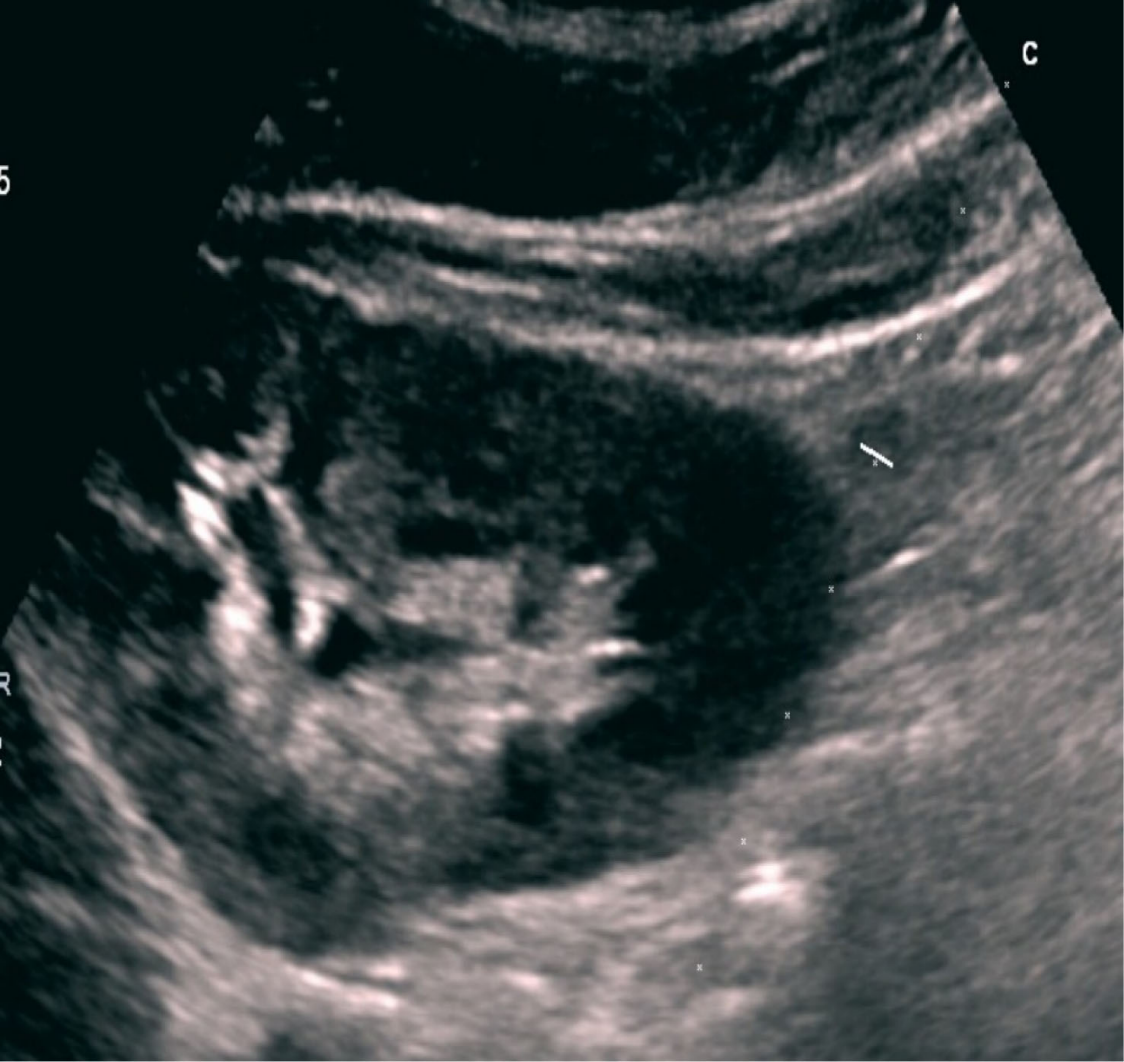
Ultrasound-guided needle placement (right kidney lower pole).

**Figure 3:**
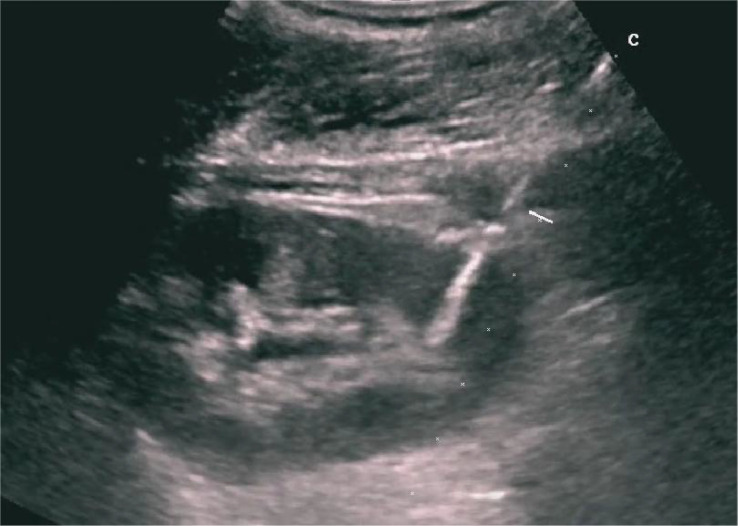
Ultrasound-guided display of the needle.

**Figure 4:**
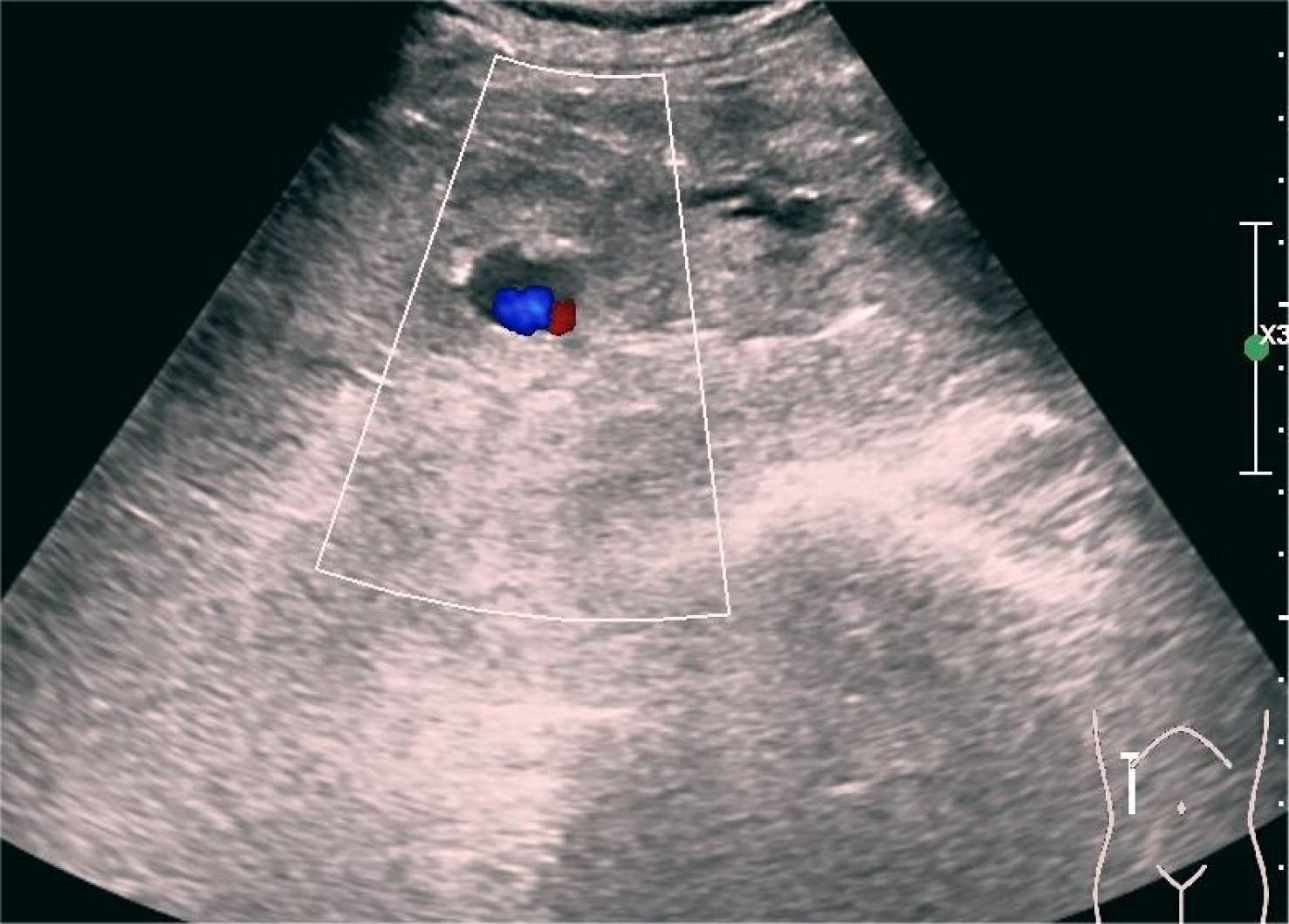
Post-puncture color Doppler ultrasound revealing hemorrhagic foci (transverse section).

**Figure 5:**
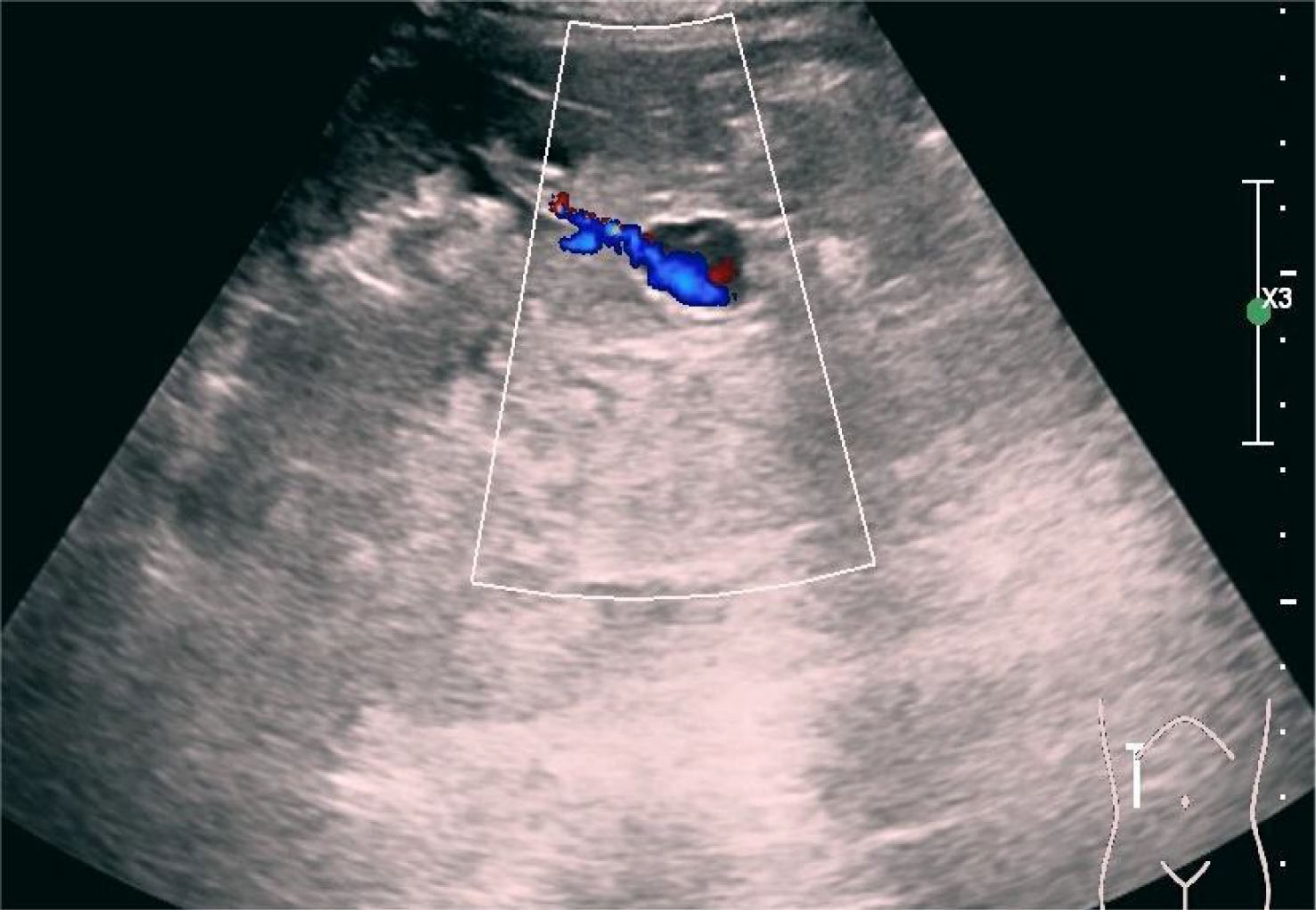
Color Doppler ultrasound after puncture revealing hemorrhagic foci (longitudinal section).

**Figure 6:**
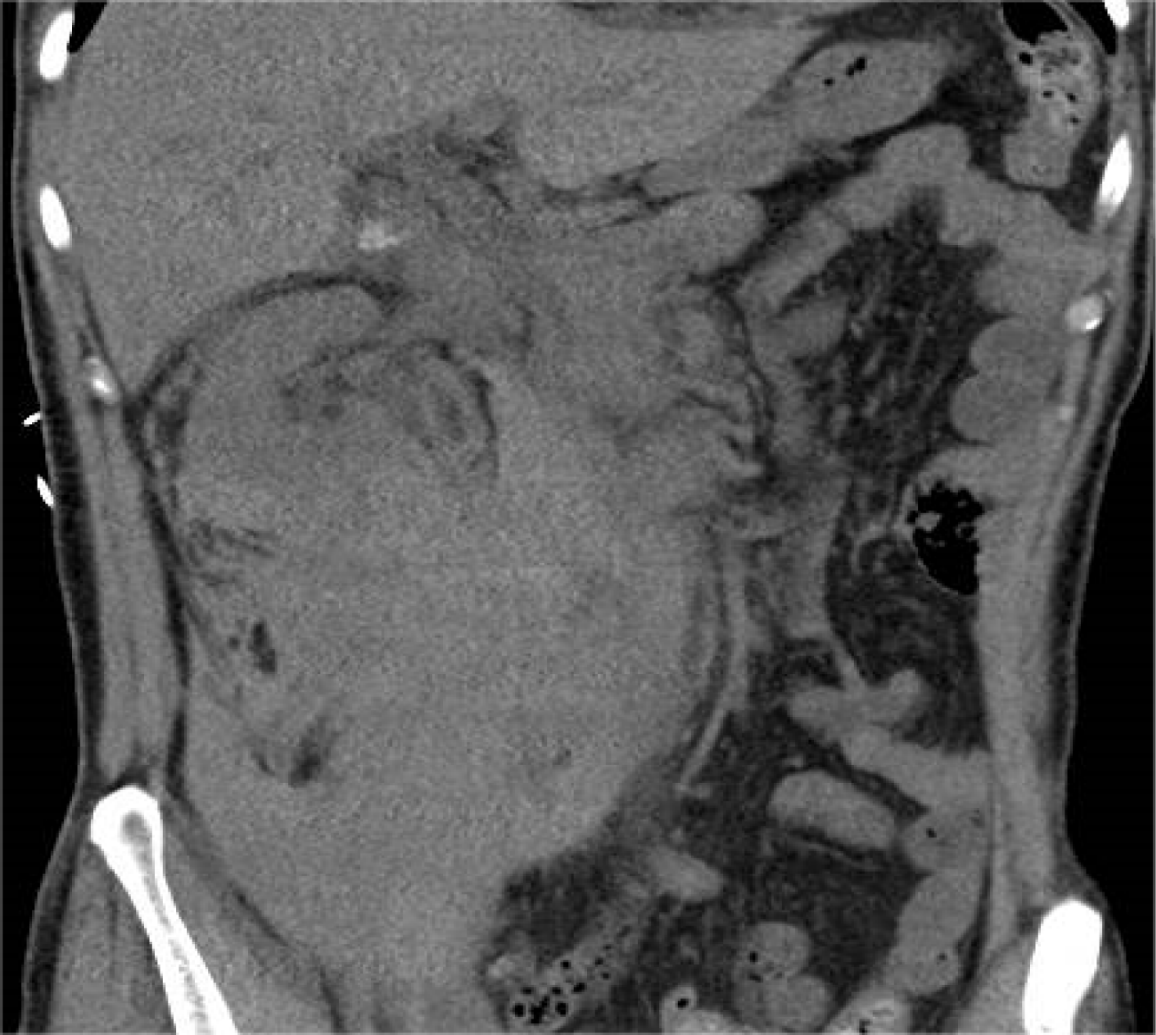
CT coronal section revealing a perirenal hematoma on the right kidney.

**Figure 7:**
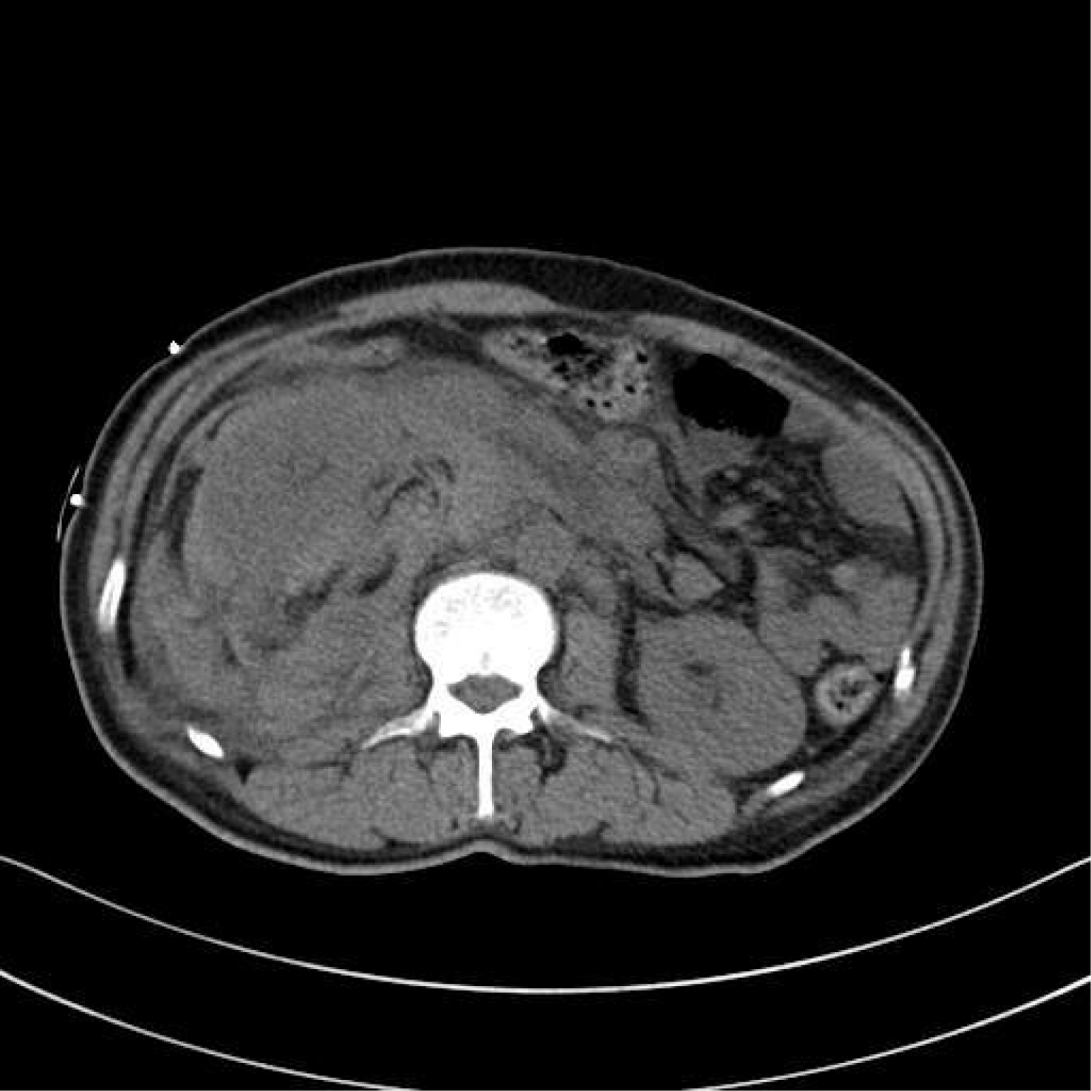
CT cross-sectional images revealing a perirenal hematoma on the right kidney.

**Figure 8:**
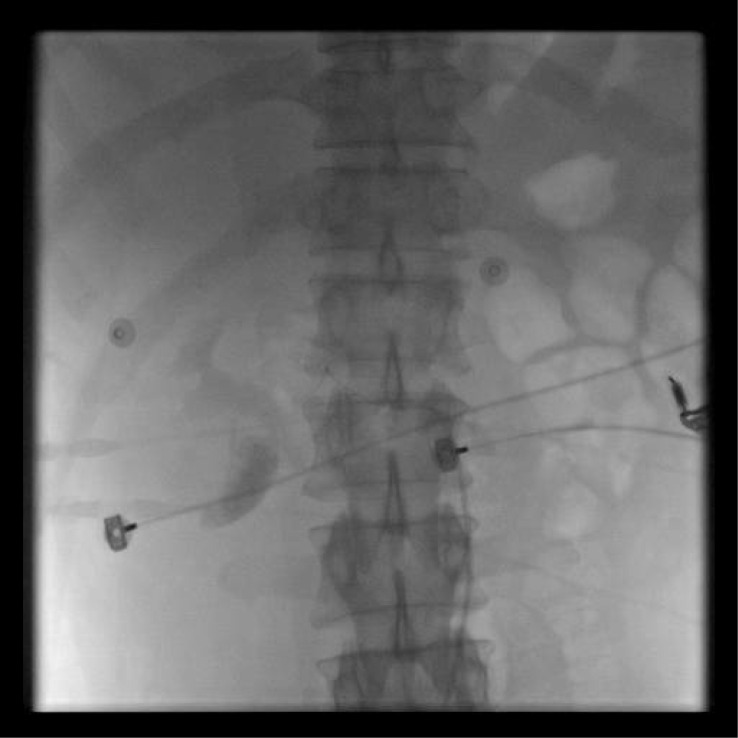
Abdominal plain radiograph revealing a right renal arteriovenous fistula.

**Figure 9:**
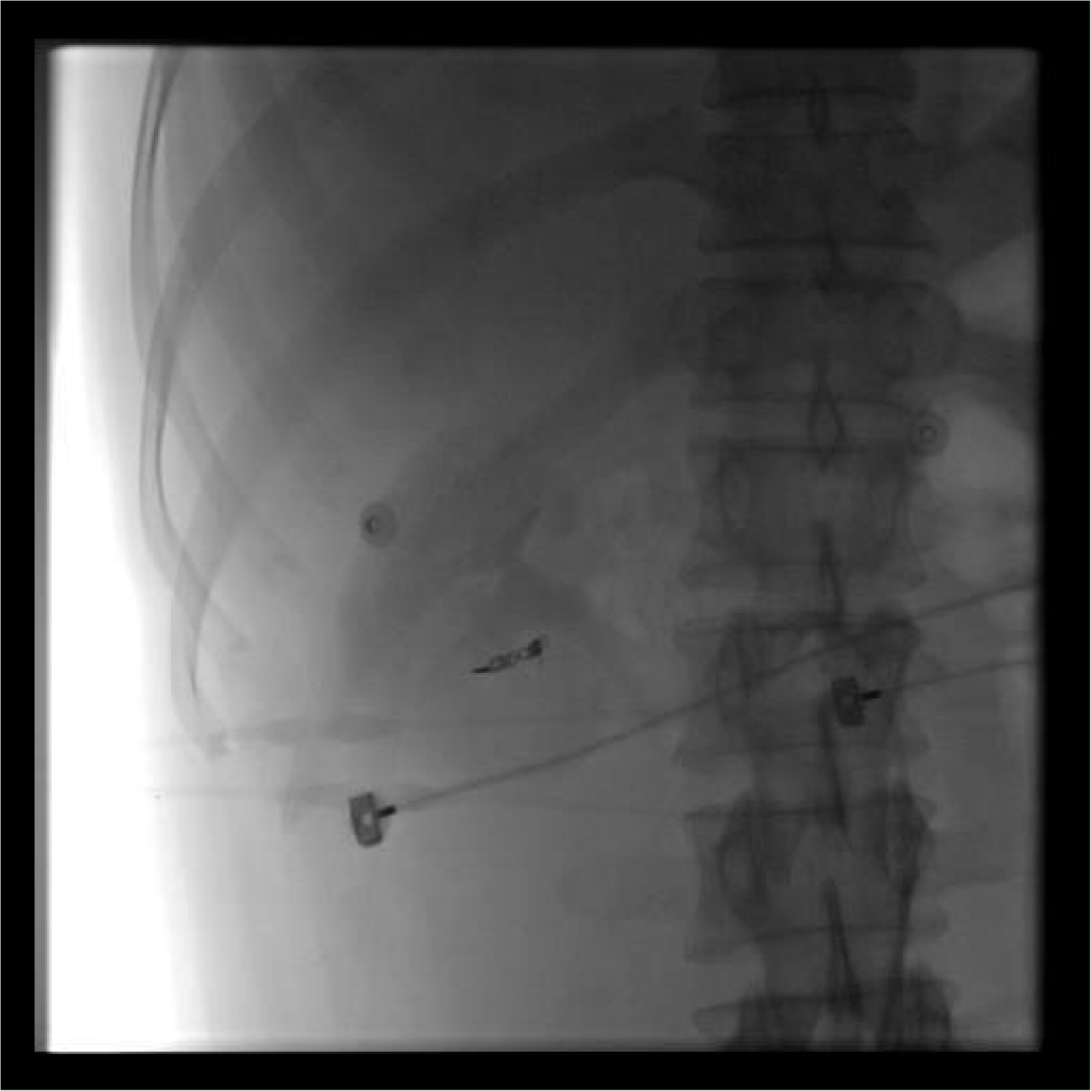
Post-interventional treatment of the right renal arteriovenous fistula on abdominal plain radiograph.

## Discussion

In recent years, the prevalence of chronic kidney disease has shown an increasing trend, severely compromising human health and becoming a public health issue of global concern (Huang et al., 2022). Percutaneous renal biopsy (PRB) guided by color Doppler ultrasound is a commonly used diagnostic method and is an important tool for clinicians, providing effective diagnostic evidence for the diagnosis, evaluation, and treatment of kidney diseases. This procedure has now become the “gold standard” for diagnosing kidney diseases (Tian et al., 2022). Post-biopsy bleeding is the primary risk associated with PRB, and severe cases may be life-threatening (Zhang et al., 2021).

PRB under color Doppler ultrasound guidance is a commonly used procedure in ultrasound-guided interventions and receives significant attention from clinical departments in daily diagnostic and therapeutic work. As ultrasound-guided interventions are invasive procedures, they often carry certain surgical risks and complications. PRB is now widely applied in the clinical diagnosis and treatment of kidney diseases (Haochen et al., 2019). Studies indicate that the incidence of bleeding following PRB reaches as high as 58.5% (Li et al., 2020), significantly exceeding the 0.9%–5.3% reported in the existing literature (Feldmann et al., 2018). The most severe bleeding complications can result in renal AV fistula formation, leading to massive hemorrhage, hemorrhagic shock, and life-threatening conditions. Postoperative hemorrhage remains the primary complication causing mortality following interventional procedures. Renal AV shunt, a rare pathologic condition, is classified into two categories—traumatic and non-traumatic—and can cause massive hematuria, retroperitoneal hemorrhage, pain, and high-output heart failure. Although transcatheter embolization is a less invasive and an effective treatment option, it has a potential risk of complications, including renal infarction and pulmonary embolism, and a potential risk of recanalization. The successful embolization of renal AV shunt requires complete occlusion of the shunted vessel while preventing the migration of embolic materials and preserving normal renal arterial branches, which is dependent on the selection of adequate techniques and embolic materials for individual cases based on the etiology and imaging angioarchitecture of the renal AV shunt (Miyuki et al., 2016).

PRB provides important information for assessment of the diagnosis, management, and prognosis of patients with renal disease. Complications following renal biopsy have decreased thanks to improvements in the imaging techniques and biopsy needles. Korbet et al. investigated complications of renal biopsy in patients with native kidneys. Death or major complications that required interventions, such as transfusion of blood products or invasive radiologic or surgical procedures, occurred in 6.6% of biopsies. Among these major complications, 57% were identified by 4 h after biopsy, while 72% were detected by 8 h, 89% by 24 h, and 11% after 24 h (Korbet et al., 2014). Simard-Meilleur et al. reported that complications developed in 84% of affected patients by 8 h, increasing to 86% at 12 h and 94% at 24 h (Simard-Meilleur et al., 2014). Thus, major complications are considered to be uncommon from 24 h after PRB. However, little has been reported about the occurrence of late complications from 24 h (Takeuchi et al., 2018).

Numerous adverse factors contribute to bleeding following PRB under color Doppler ultrasound guidance. These primarily include elevated BP, thin lower pole renal parenchyma, low platelet counts, abnormal renal function, coagulation disorders, anemia, more than three punctures, mismatched biopsy needle gauge, poor patient cooperation, and operator inexperience. Research indicates that advanced age, hypertension, renal insufficiency, and low platelet counts are all closely associated with hematoma and bleeding following PRB (Xu et al., 2022).

Strengthening the prevention and control of complications across preoperative preparation, procedural techniques, and postoperative care is essential to ensure effective management of complications following PRB. This approach reduces the incidence of postoperative bleeding, thereby enhancing the safety and efficacy of PRB in clinical practice (Cheng et al., 2023). Rigorous quality control throughout the preoperative, intraoperative, and postoperative phases is essential to minimize complications and enhance the safety of interventional procedures. Statistical analysis of the causes leading to bleeding after color Doppler ultrasound-guided PRB, implementation of bleeding prevention measures, improvement of the procedural proficiency, and training patients in respiratory coordination before surgery are all critical. Preoperative assessment, including BP, coagulation function, platelet counts, lower pole renal parenchymal thickness, and renal function, should be strengthened. There should be strict adherence to indications and contraindications for interventional puncture. Procedural precision during intervention should be improved by carefully controlling the puncture angle and depth, minimizing the number of punctures, and enhancing postoperative observation and nursing care to promptly identify complications.

In summary, following ultrasound-guided renal biopsy, close postoperative monitoring and nursing care enable rapid detection of complications via ultrasound imaging. This facilitated prompt notification to the clinical team for early, proactive, and effective treatment. In this case, postoperative bleeding, massive hematoma, and renal AV fistula were promptly identified through clinical postoperative care protocols. Emergency measures were immediately initiated, including interventional embolization for acute hemorrhage, preventing severe adverse outcomes such as life-threatening hemorrhage.

## Data Availability

The data presented in this case report consists of de-identified clinical records and imaging data, which are stored in the hospital’s internal secure database and are not publicly available due to patient privacy regulations. No new raw data were generated in this study. All findings are included in the article. For inquiries related to data access, please contact the corresponding author.
